# Electrodeposited Magnetic Nanowires with Radial Modulation of Composition

**DOI:** 10.3390/nano12152565

**Published:** 2022-07-26

**Authors:** Claudia Fernández-González, Alejandra Guedeja-Marrón, Beatriz L. Rodilla, Ana Arché-Nuñez, Rubén Corcuera, Irene Lucas, María Teresa González, Maria Varela, Patricia de la Presa, Lucía Aballe, Lucas Pérez, Sandra Ruiz-Gómez

**Affiliations:** 1 Instituto Madrileño de Estudios Avanzados—IMDEA Nanociencia, 28049 Madrid, Spain; clafer03@ucm.es (C.F.-G.); beatriz.rodilla@imdea.org (B.L.R.); ana.arche@imdea.org (A.A.-N.); teresa.gonzalez@imdea.org (M.T.G.); 2 Departamento de Física de Materiales, Universidad Complutense de Madrid, 28040 Madrid, Spain; aguedeja@ucm.es (A.G.-M.); mvarela@fis.ucm.es (M.V.); pmpresa@ucm.es (P.d.l.P.); 3 Instituto de Nanociencia y Materiales de Aragón (INMA), Universidad de Zaragoza—-CSIC, Mariano Esquillor, Edificio I+D, 50018 Zaragoza, Spain; 744439@unizar.es (R.C.); ilucas@unizar.es (I.L.); 4 Departamento Física de la Materia Condensada, Universidad de Zaragoza, Pedro Cerbuna 12, 50009 Zaragoza, Spain; 5 Instituto de Magnetismo Aplicado, 28230 Las Rozas, Spain; 6 Alba Synchrotron Light Facility, Carrer de la Llum 2-26, 08290 Cerdanyola del Valles, Spain; laballe@cells.es; 7 Surface Science and Magnetism of Low Dimensional Systems, UCM, Unidad Asociada al IQFR-CSIC, 28040 Madrid, Spain; 8 Max-Planck-Institut für Chemische Physik fester Stoffe, 01187 Dresden, Germany

**Keywords:** core–shell nanowires, nanomagnetism, electrodeposition

## Abstract

In the last few years, magnetic nanowires have gained attention due to their potential implementation as building blocks in spintronics applications and, in particular, in domain-wall- based devices. In these devices, the control of the magnetic properties is a must. Cylindrical magnetic nanowires can be synthesized rather easily by electrodeposition and the control of their magnetic properties can be achieved by modulating the composition of the nanowire along the axial direction. In this work, we report the possibility of introducing changes in the composition along the radial direction, increasing the degrees of freedom to harness the magnetization. In particular, we report the synthesis, using template-assisted deposition, of FeNi (or Co) magnetic nanowires, coated with a Au/Co (Au/FeNi) bilayer. The diameter of the nanowire as well as the thickness of both layers can be tuned at will. In addition to a detailed structural characterization, we report a preliminary study on the magnetic properties, establishing the role of each layer in the global collective behavior of the system.

## 1. Introduction

Since the discovery of giant magnetoresistance [1,2], magnetic multilayers have played a key role in the advance of nanomagnetism and spintronics [3]. In multilayered systems, the thickness of the different layers [4], as well as interfacial effects [5,6], control the coupling between the different magnetic layers, governing the collective magnetic behavior of the whole structure and giving rise to the stabilization of different magnetic textures [7,8,9]. Most of the studies and applications regarding nanomagnetism, spintronics and interfacial effects have been mainly developed in planar structures, typically with lithographed patterns. However, in the last years, 3D magnetic systems [10] and curvilinear nanostructures [11] have emerged as an advanced alternative for the study of novel nanomagnetic effects and the development of a new generation of spintronics applications.

Nanowires (NWs) are among the most studied curvilinear systems [12,13,14]. In these systems, changes of composition along the axial direction, i.e., synthesizing multilayers along the growth direction, can be used to tune the magnetic properties [15,16] or to stabilize topologically complex domain structures [17,18]. By appropriately choosing the thickness of consecutive multilayers, a magnetization ratchet in the magnetization dynamics can also be produced [19]. All the works on NWs have been focused on tailoring the composition along the axial direction. Adding the possibility of modulating the composition along the radial direction, preparing core–shell and radial nanostructured NWs, can significantly expand the possibilities of these structures.

Template-assisted electrodeposition is one of the most efficient techniques to grow one-dimensional metallic nanostructures [20,21]. The composition of these nanostructures can be modulated along the radial direction, preparing core–shell nanostructures using different approaches. On the one hand, once grown, the surface of metallic NWs can be oxidized to produce metal/oxide core/shell structures [22,23]. Although easy to implement, this approach clearly limits the selection of materials forming the structure and does not allow the growth of more than one layer in the radial direction. On the other hand, an electroless approach has also been used to coat ferromagnetic metallic NWs with a Au shell [24,25], with the focus on the development of biomedical applications. Finally, different chemical methods can also be used to grow a tube covering the nanoporous template and afterward filling the pore by electrochemical deposition [26]. Atomic layer deposition (ALD) allows high accuracy in the control of the coating thickness. This technique is mostly used in the deposition of binary compounds such as oxides, nitrides, carbides, etc. [27,28,29], although magnetic metals such as Ni and Co can also be synthesized [30]. Despite the fact that this technique is quite versatile, it does not easily allow the synthesis of nanostructures with several layers along the radial direction.

In this work, we present a novel approximation based on multi-step template-assisted electrodeposition in order to modulate the composition along the radial direction in ferromagnetic NWs. The proposed electrodeposition methodology allows the growth of metallic structures with a different composition and number of layers. To illustrate the method, we have synthesized ordered nanostructures on a flexible metallic layer consisting of trilayers of FeNi/Au/Co and Co/Au/FeNi. In these magnetic structures, the coupling between both magnetic layers depends on the thickness of the Au layer. In addition to a detailed structural characterization, we report a preliminary study on the magnetic properties of the system.

## 2. Materials and Methods

Core–shell nanostructures have been synthesized by template-assisted electrochemical deposition. Nanoporous polycarbonate membranes purchased from Whatman (GE Healthcare, Chicago, IL, USA) have been used as templates, with a diameter of 50 nm, thickness of 6 μm and a pore density of $6\times 10^{8}$ cm${}^{-2}$. Electrodeposition was carried out in a three-electrode electrochemical cell, using a Pt mesh as counter electrode and a Ag/AgCl (3 M NaCl) electrode as reference electrode. All voltage values in this work are referred to this reference electrode. The electrodeposition was controlled and monitored with a PGSTAT potentiostat (Metrohm-Autolab, Utrecht, The Netherlands). Before electrodeposition, a 100 nm thick Au film was thermally evaporated on one side of the membrane to act as working electrode.

Permalloy (FeNi) NWs were grown at −1.5 V from an electrolyte at room temperature composed by 0.8 M NiSO${}_{4}$, 0.02 M NiCl${}_{2}$, 0.16 M FeSO${}_{4}$ and 0.4 M H${}_{3}$BO${}_{3}$. Under these conditions, the growth rate of the FeNi nanowires is ∼200 nm/s. In the case of the growth of the FeNi external layer, the electrolyte was diluted in water in the proportion 1:10 to reduce the growth rate of the shell down to 20 nm/s. The pH of the solutions was adjusted to 2.3 using H${}_{2}$SO${}_{4}$ 10% vol. For the growth of Co NWs, an electrolyte composed of 0.5 M CoSO${}_{4}$ and 0.5 M H${}_{3}$BO${}_{3}$ was used. The Co layer was grown using a bath composed of 0.01 M CoSO${}_{4}$ and 0.1 M H${}_{3}$BO${}_{3}$. In both cases, the applied voltage was −1.1 V and the pH was adjusted to 2.3 using H${}_{2}$SO${}_{4}$ 10% vol. In this case, the growth rates for the Co nanowires and shells are 50 nm/s and 1 nm/s, respectively. All chemicals were of analytical grade, and they were used without further purification and mixed in deionized water. For Au electrodeposition, an Orosene commercial electrolyte (ORE+4, Italgalvano, Lodi Vecchio, Italy) was used. While FeNi and Co were synthesized under applying constant potential, pulse plating was used for the growth of the Au layers to reduce the stress generated during the Au deposition. Growth pulses (V${}_{\mathrm{on}}-1.5$ V and t${}_{\mathrm{on}}=0.1$ s) were combined with rest pulses (V${}_{\mathrm{off}}=-1.5$ V and t${}_{\mathrm{off}}=$1 s).

A scanning electron microscope (SEM) JEOL JSM 6335F (Tokio, Japan) equipped with an energy-dispersive X-ray (EDX) microanalysis system was used to study the morphology and composition of the wires. All SEM-EDX measurements were performed in areas with high density of NWs. Therefore, the compositions reported here are averaged laterally over large amounts of NWs. SEM images were also used to estimate the length of the nanowires. For that, the NWs were sonicated to detach them from the substrate and deposited flat by drop-casting on top of a silicon substrate. The thickness of the different layers as well as local composition of selected NWs were studied in individual core–shell NWs with high-resolution scanning transmission electron microscopy (STEM) measurements. The observations were carried out in a JEOL ARM200cF (Tokio, Japan) at 200 kV at Centro Nacional de Microscopía Electrónica (CNME) at the University Complutense of Madrid. The microscope is equipped with a CEOS spherical aberration corrector and a Gatan Quantum electron energy-loss spectrometer (EELS). Specimens were prepared by drop-casting method on a carbon-coated copper grid. Random noise was removed from EELS data using principal component analysis. EELS compositional maps were produced by subtracting the background using a power-law fit followed by integration of the signal below the relevant edges. Before any electron and X-ray microscope characterization, the NWs were released from the gold layer by few minutes of sonication in ethanol.

Transmission X-ray Microscopy (TXM) measurements were performed at the MISTRAL beamline of the ALBA Synchrotron, using soft X-rays from a bending magnet source [31,32]. A capillary condenser after the monochromator exit slit focuses the radiation to the sample, which is installed on a goniometer and on an x-y-z translation stage. After the sample, a Fresnel zone plate with outermost zone width of 25 nm acts as objective lens of the microscope, generating a ∼×1600 magnified image on a direct illumination CCD detector. The NWs were sonicated and deposited flat by drop-casting method on top of an X-ray transparent SiN membrane, which was then installed at the microscope.

Magnetic hysteresis loops were measured with a MPMS-5S SQUID magnetometer (Quantum Design, San Diego, CA, USA). The magnetic signal from the samples was in all cases corrected from the diamagnetic background of the sample holder. To support the experimental observations, micromagnetic simulations were performed with the mumax${}^{3}$ code. The total length of the cylindrical NWs was fixed to 0.5 μm and the diameter was set to 50 nm for the core, 5 nm and 15 nm for the Au layer and 5 nm, 10 nm and 30 nm for the outer layer. Finite element discretization size was chosen to be 4 nm for both layers. For both types of layers, we consider a structure without magnetocrystalline anisotropy. The exchange constant was fixed at A${}_{ex}$ = 13 × 10${}^{-12}$ J/m and the damping constant at α = 0.02 for both FeNi and Co. The saturation magnetization was set to $\mu_{0}$M${}_{S}$ = 0.8 T for the Permalloy and $\mu_{0}$M${}_{S}$ = 1.76 T for the Co.

## 3. Results and Discussion

The magnetic nanostructures synthesized in this work were arrays of FeNi/Au/Co and Co/Au/FeNi NWs grown on top of flexible Au substrates. We have chosen FeNi and Co for the two magnetic layers because these materials are expected to have different coercivity, which make it easier to distinguish the effect of the coupling of the inner and outer layers on the global magnetic properties of the system. In addition, having different materials in the core and shell allows to study both layers independently, using synchrotron radiation-based techniques such as TXM.

**Figure 1:**
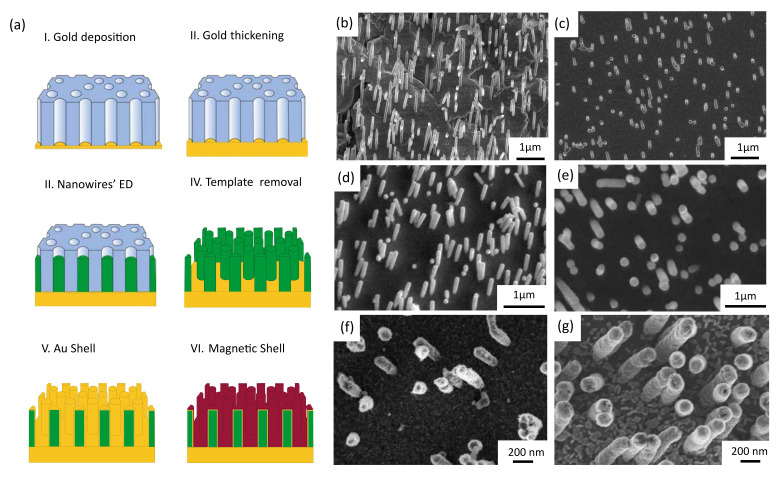
(**a**) Scheme of the steps followed to grow core–shell NWs. (**b**–**g**) SEM images of the samples at different stages of elaboration: FeNi (**b**) and Co (**c**) NWs after dissolution of polycarbonate membrane. FeNi (**d**) and Co (**e**) NWs covered by a shell of Au. FeNi/Au (**f**) and Co/Au (**g**) NWs after the growth of the Co and FeNi shell, respectively.

Figure 1a summarizes the growth procedure, starting with porous polycarbonate membranes as the templates with one side coated with a 100 nm Au layer (I). In order to use this layer as a working electrode, its thickness was increased from 100 nm to 1 μm using a series of voltage pulses as described in reference [33]. After that, FeNi (Co) NWs were electrodeposited in the pores of the membrane (III). The growth time is adjusted to obtain NWs with a length of 1 μm approximately. By performing cleaning cycles of dichloromethane, acetone and propanol, the polycarbonate template was removed, leaving the samples as a network of vertical free-standing NWs attached to the flexible Au base (IV). The network of NWs was then used as an electrode for the following electrodeposition steps of the Au layer (V) and the magnetic Co (FeNi) outer layer (VI).

The morphology of the NWs after the different growth steps was explored by SEM (see Figure 1). Figure 1b,c correspond to FeNi and Co vertical NWs, respectively, after the removal of the polycarbonate template (step IV in Figure 1a). From the image, the random distribution of the NWs is clear, reflecting the distribution of the nanopores of the template. The NWs are homogeneous in diameter (50 nm) with lengths around 1 μm and remain vertical without evidence of agglomeration after removing the template. The distance between the NWs allows the deposition of a homogeneous Au shell fully covering the surface of the previously grown NWs (see Figure 1d,e). Finally, Figure 1f,g show the final morphology of the NWs, after growing the Co and FeNi outer layers (step VI). The final diameter has increased to approximately 100 nm after the deposition of the different layers, but the wires are homogeneous in diameter along their length and the material is completely covering the surface.

**Figure 2:**
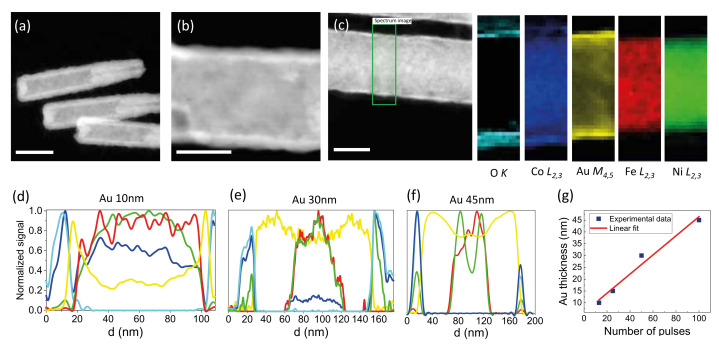
STEM-EELS characterization of the NWs. (**a**,**b**) High-angle annular dark-field images of the nanostructures at low and high magnification. (**c**) EELS spatial compositional maps showing the O, Au, Co, Fe and Ni content within the region marked with a green rectangle in the image. (**d**–**f**) From left to right, EELS line scans showing the elemental profiles for different samples with increasing Au shell thickness. (**g**) Calibration curve of the Au shell thickness as function of the number of repetitions of on/rest pulses. The scale bar is 50 nm for all images.

TEM measurements provide insight into the structure of the NWs, together with a calibration of the thickness and a check of the homogeneity of the different layers. We have investigated FeNi/Au/Co NWs with different Au thicknesses of 10 nm, 30 nm and 45 nm. Figure 2a,b show high-angle annular dark-field (HAADF) images of the NWs. In both cases, it is clear that the morphology of the NW is preserved after growing all the layers. The Co and Au layers are homogeneously coating the inner FeNi NW with a slight increase in roughness due to the different electrodeposition processes. The radial change in the composition can be better seen in a quantification of the EEL spectra. Figure 2c shows the element maps measured by the EELS in the rectangular area marked on the NW, based on the analysis of the O *K*, Co $L_{2,3}$, Au $M_{4,5}$, Fe $L_{2,3}$ and Ni $L_{2,3}$ peaks, from the left to the right. The distribution of the elements clearly shows an FeNi core with the thickness in the range of 70 nm, surrounded by a homogeneous 10 nm thick Au layer and a 15 nm thick outer Co shell. There is also some oxygen in this Co external layer, most likely due to the thermal oxidation of the Co layer during the time between the growth and the preparation for the TEM measurements. Finally, a comparison of the NWs with different Au thicknesses can be extracted from the composition measured in line scans across the diameter (Figure 2d–f). All wires have the same FeNi core (approximately 70 nm), as shown by the evolution of the Fe and Ni lines. However, when comparing the distribution of Au, there is a clear difference between the samples, being the thickness of the Au layer at 10 nm (d), 30 nm (e) and 45 nm (f). Figure 2g shows the thickness of the Au shell extracted from the experimental measurements as a function of the number of voltage pulses used for the synthesis of the Au intermediate layer. There is a clear linear dependence, demonstrating that the proposed growth protocol allows for an accurate control of the layer thickness.

**Figure 3:**
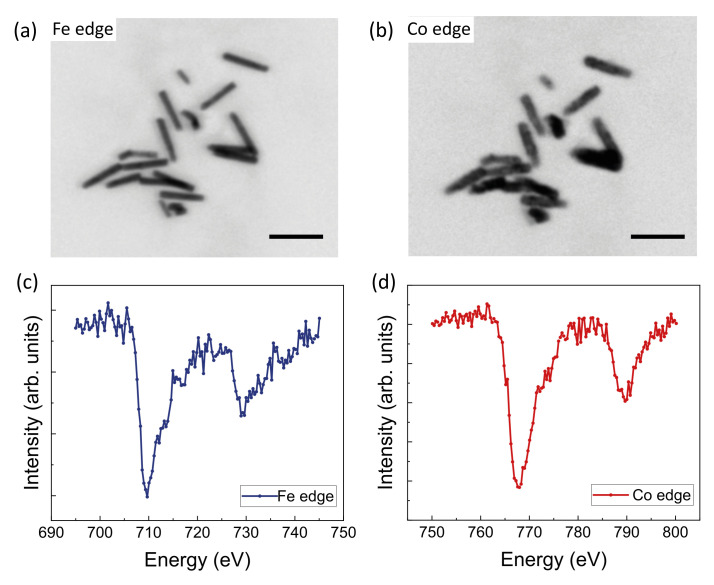
X-ray Transmission Microscopy characterization of the NWs. (**a**,**b**) Absorption images at the Fe $L_{3}$ and Co $L_{3}$ edges (**c**,**d**) and X-ray absorption spectra (XAS) of Fe and Co absorption edges of a cluster of FeNi/Au/Co NWs. The scale bar is 500 nm in both images.

Figure 3 shows the TXM images at the Fe $L_{3}$ and Co $L_{3}$ absorption edges of a cluster of NWs, where the Fe core and Co shell are clearly distinguished. The absorption spectra at the Fe and Co *L* edges are shown in Figure 3c,d. The spectra correspond to Fe and Co that are predominantly metallic with a slight shoulder at higher photon energy in the case of Co, revealing the partial oxidation of the outermost (surface) layer.

**Figure 4:**
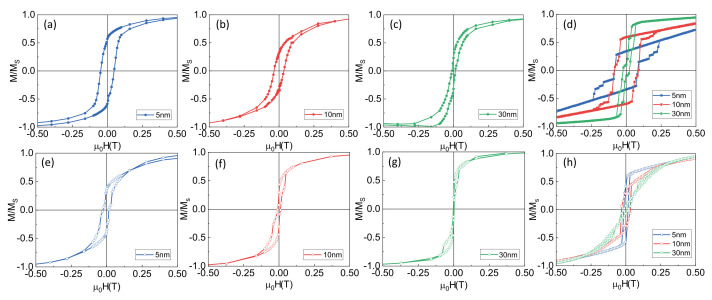
Influence of the thickness of the external magnetic layer in the hysteresis loops for FeNi/Au(5 nm)/Co NWs. Hysteresis loops measured with the field applied parallel (panels (**a**–**c**)) and perpendicular to the longitudinal axis of the NWs (panels (**e**–**g**)). Panels (**d**) and (**h**) correspond to micromagnetic simulations of the magnetic external layer.

The control of the thickness of the different layers allows for the tuning of the magnetic properties of the NWs. Figure 4 shows the hysteresis loops measured at room temperature in arrays of NWs with an FeNi core, a non-magnetic 15 nm thick Au spacer and a Co outer layer with different thicknesses (5 nm, 10 nm and 30 nm, respectively), together with micromagnetic simulations of the magnetic behavior of the external magnetic shell. All the samples show an easy axis along the direction of the longitudinal axis of the NW, as expected from the contribution of the shape anisotropy. In the case of the hysteresis loops measured with the applied field along the longitudinal axis (Figure 4a–c), there is a clear reduction in the coercivity when the thickness of the layer increases. This effect is the combination of two different phenomena: On the one hand, a thicker layer favors the demagnetizing processes. On the other, the magnetostatic interaction between NWs becomes higher for thicker magnetic shells, which also favors the reversal of magnetization at lower magnetic fields. When applying the field in the direction perpendicular to the NWs, there are contributions from the NWs—the field is applied along a hard axis—as well as from the Co thin film grown on the bottom of the arrays—the field is applied along an easy axis. In this case, the hysteresis loops are the combination of both contributions, an effect which is clear for all Co thicknesses (Figure 4e–g).

We have carried out micromagnetic simulations for a better understanding of the system. In particular, we have simulated the magnetic behavior of the magnetic shell to elucidate its contribution. Figure 4d,h show the hysteresis loops for an array of seven non-magnetic NWs covered by a magnetic shell, for the magnetic field applied along the longitudinal axis and perpendicular to it, respectively. Firstly, from the simulation, it is clear that the easy axis is parallel to the longitudinal axis of the NWs, as expected. For the applied field along the easy axis, an increase in the thickness reduces the coercivity, as seen in the experimental results. In addition, the intermediate states with the magnetization lower than the saturation can be found for fields close to the coercive field. They correspond to the switching of individual NWs, showing antiparallel orientation to the applied field due to the magnetostatic interaction between the NWs. The jumps in the hysteresis loops are not observed in the experimental measurements because it is not possible to distinguish the individual switching of each NW in the global behavior of the system, but these reversal processes are behind the decrease in the coercivity with increasing thickness. The reversal of the magnetization starts with the rotation of the magnetization in the thin film covering the gold substrate. Afterward, the cylindrical shells start switching their magnetization when the applied field is reversed, and once all of them are aligned along the direction of the applied field, the thin film ends its rotation.

When applying the field perpendicular to the NWs, the reversal of the magnetization starts in the NWs, until their magnetization is oriented along their long axis. Then, the thin film starts rotating at the same time that magnetic vortices are generated on the top part of the cylinders. When the thin film is oriented in the direction of the magnetic applied field, the magnetization in the NWs goes in the direction of the applied field. The experimental measurements are more similar to the simulations for the thicker outer layers because the contribution of the ferromagnetic core behaves, in this case, similar to the shell due to the coupling between both, increasing the contribution of the NWs to the global magnetization processes.

**Figure 5:**
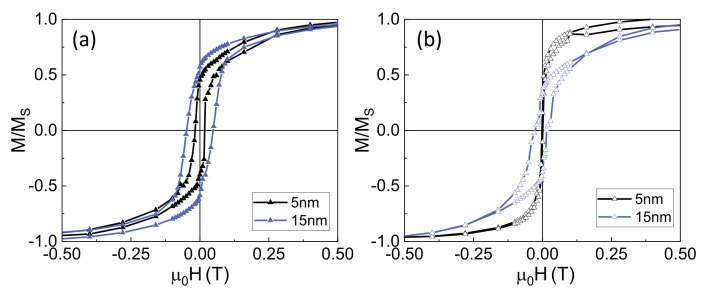
Influence of the thickness of the Au layer on the magnetic properties of FeNi/Au/Co arrays with 5 nm thick Co. Hysteresis loops measured with the field applied (**a**) parallel and (**b**) perpendicular to the longitudinal axis of the NWs.

We have also studied the possibility of controlling the magnetic properties of the system by tuning the thickness of the non-magnetic Au spacers. Figure 5 shows the hysteresis loops of two different Py/Au/Co systems in which the thickness of the Co layer has been kept constant (in 5 nm), whereas the thickness of the Au layer has been changed from 5 nm to 15 nm. Figure 5a shows the hysteresis loops of both samples with the magnetic field applied along the longitudinal axis. There is a clear reduction in the coercivity when reducing the Au thickness: the coupling between the FeNi NW and the Co outer shell allows the reversal of the latter for a lower magnetic field. The coupling between both layers is also clearly shown when applying the field along the direction perpendicular to the longitudinal axis of the NWs (Figure 5b) in which only one magnetization process is shown, corresponding to a hard magnetization axis. When increasing the thickness of the Au layers, the behavior of the different layers emerges.

As illustrated by this example, the modulation of the composition along the radial direction introduces an additional possibility for tailoring the magnetic behavior of the magnetic nanowires that can be explored in magnetic and spintronics devices. In addition, this possibility of coating metallic nanowires with external layers may enlarge the potential applications of nanowires, for example, producing Au biocompatible coatings in electrodeposited nanowires for biomedical [22,34,35] or sensing [36] applications. Finally, providing that the nanowires are standing up on a flexible substrate with a total large active area, these core–shell structures may be designed to have applications in catalysis [37], neural applications [33,38,39] or flexible devices [40,41,42], among others.

## 4. Conclusions

In this work, we have presented a novel approximation based on multi-step template-assisted electrodeposition that allows the fabrication of NWs with a radial modulation of composition, with the following advantages: (i) the ability to combine materials with different physical properties, (ii) the possibility of introducing a non-magnetic layer, and (iii) the freedom to tune the geometrical properties (the diameter of the core, thickness of the shell and separation between them). The templates define the characteristics of the core (the diameter and separation between wires) while the time of the electrodeposition process defines the thickness of the shells. Following this approximation, the material of the core, shell and intermediate layer can be chosen independently, and the thickness of each individual layer can be tuned. We have also shown that this control of the morphology allows the tuning of the magnetic properties.

## Data Availability

The data presented in this study are available on request from the corresponding authors.

## Sample Availability

The nanowires described in this manuscript are available from the authors upon reasonable request.
